# Relation of the Use of Coffee to Susceptibility to Typhoid Fever

**Published:** 1888-07

**Authors:** W. A. Cusick

**Affiliations:** Oregon


### RELATION OF THE USE OF COFFEE TO SUSCEPTIBILITY TO TYPHOID FEVER.

BY W. A. CUSICK, M. D., OF OREGON.

I have read in April number of RECORD, p. 148, observations by Dr. H. S. Herr, as to the possible relation of the use of coffee to typhoid fever. The subject is possessed of more than ordinary interest to me, as I have made similar observations to those of Dr. Herr, and am convinced that while coffee may not be an antiseptic proper, it is unquestionably an antiferment, and (where rightly prepared) has salutary effect on the intestinal tract by preventing gaseous formations, and stimulant, and tonic effect upon the nerve filaments or "end organs," it becomes a corrector of intestinal secretions, and in this way, to some extent, at least, may be considered a preventive of typhoid fever. I will say in this connection, that in a practice of twenty-five years, I have not observed a fatal issue in any case of typhoid fever, which I can call to mind, where the patient expressed a fondness for coffee and used it freely during the course of the sickness, and I have, as a consequence, for some years past, encouraged my typhoid patients to use coffee freely unless they expressed an absolute dislike for it, or there had been a previous idiosyncrasy interdicting its use.

I regard coffee as much more than a simple stimulant, indeed, as an actual food, and believe, from observation, that it imparts "staying qualities" to the nervous system, which in self-limited fevers, will often tide the patient over the critical period to convalescence when he would otherwise sink and die.

